# Continuous Self-Cycling Fermentation Leads to Economical Lycopene Production by *Saccharomyces cerevisiae*

**DOI:** 10.3389/fbioe.2020.00420

**Published:** 2020-05-15

**Authors:** Zhiming Wang, Xiangyu Li, Chao Yu, Shuhuan Lu, Shuting Xiong, Yingjin Yuan

**Affiliations:** ^1^Frontier Science Center for Synthetic Biology and Key Laboratory of Systems Bioengineering (Ministry of Education), School of Chemical Engineering and Technology, Tianjin University, Tianjin, China; ^2^SynBio Research Platform, Collaborative Innovation Center of Chemical Science and Engineering, Tianjin, China; ^3^CABIO Biotechnology (Wuhan) Co., Ltd., Wuhan, China; ^4^Hubei Province Nutrition Chemicals Biosynthetic Engineering Technology Research Center, Wuhan, China

**Keywords:** lycopene, *Saccharomyces cerevisiae*, wastewater, biomass residue, self-cycling fermentation

## Abstract

The economic feasibility and waste treatment problem are challenges to the industrialization of lycopene production from *Saccharomyces cerevisiae*. In this study, fermentation wastewater, biomass residue, and residual D-galactose are recycled for lycopene production. Results show that when fresh water is totally replaced by wastewater, lycopene titer attains 1.21 ± 0.02 g/L, which is 14.2% higher than the fresh water group (*P* < *0.05*). An 80% replacement ratio of yeast extract by biomass residue causes no significant difference to lycopene production while 100% replacement ratio significantly lowers lycopene titer compared with the yeast extract group. Then, a novel fermentation medium containing wastewater and biomass residue with supplementing 3 g/L yeast extract and D-galactose is used for lycopene production. Lycopene titer increases 22.4% than the traditional fermentation in shake flasks (*P* < *0.05*). Continuous self-cycling strategy using wastewater and biomass residue was tested in shake flasks. The mean lycopene titer of the first five recycles shows no significant difference with the start batch. Scaling up to 70 L fermenter, the mean lycopene titer attains 5.88 ± 0.15 g/L in three recycles, which is 22.25% higher than the start batch (*P* < *0.05*). Economic analysis shows that the lowest unite product cost is achieved when four recycles are accomplished, which is 29.6% lower than the traditional fermentation while the chemical oxygen demand decreases 64.0%. Our study shows that continuous self-cycling fermentation process for lycopene production is feasible for the first time. The comprehensive utilization of wastewater and biomass residue from lycopene production by *S. cerevisiae* and achievement of high lycopene titer will hopefully accelerate industrialization of microbial production of lycopene.

## Introduction

Lycopene is an important carotenoid with 11 conjugated and two non-conjugated double bonds. It has attracted broad attention due to its significant benefits for human health, e.g., antioxidant activities (Suwannalert et al., 2007; Prasad and Mishra, 2014; Islamian and Mehrali, 2015), cancer prevention (Feofilova et al., 2006; Yun et al., 2008; Assar et al., 2016), and cardiovascular disease prevention (Kristenson et al., 1997; Rissanen et al., 2003; Riccioni et al., 2007). Currently, lycopene is considered to be an important ingredient in functional food and food colorant. The lycopene market is estimated to increase to more than $133 million by 2023 with an annual growth rate of 3.5% from 2018 to 2023 (IndustryARC^TM^, 2020). The total amount of tomato processing waste is reported to be 1,200,000 ton/year, in which lycopene crystal is estimated to be more than 60 ton/year, assuming that all the waste are used for lycopene extraction (Poojary and Passamonti, 2015). However, problems still exist in lycopene extraction, such as low extraction yield and requirement of high cost of instrumentation, enzymes or toxic solvents, which greatly limit lycopene production from tomato waste, thus maintains a high lycopene production cost (Saini and Keum, 2018). The other traditional way for lycopene production is chemical synthesis, which is also not able to fully meet the need of market due to food safety (Vachali et al., 2012).

Considering microbial production of high-value nutrients is more convenient and sustainable, numerous studies have engineered *Saccharomyces cerevisiae* for lycopene production and the lycopene titer increases from 1.61 g/L by Xie et al. (2015) to 1.65 g/L by Chen et al. (2016) and 2.37 g/L by Ma et al. (2019) to 3.28 g/L by Shi et al. (2019), which is the highest titer to date. Although microbial production of lycopene is successfully realized in laboratory scale, the key obstacles still hinder large-scale lycopene production, such as: low yield of lycopene, high cost of raw materials, as well as waste treatment problem. However, little attention has been paid to the wastewater and biomass residue treatment as well as cost reduction by recycling use of the wastes in these studies. Since wastewater and biomass residue would cause environment pollution and handling with wastewater and biomass residue of high chemical oxygen demand is expensive, it is quite essential to find a way out of the traditional fermentation mode.

Recycling use of wastewater and biomass residue is one of the ideal ways to solve this problem. Thus, some studies have tried to make biological waste treatment, especially recycling use of the waste to reduce waste emission and resource consumption, such as using biomass hydrolysate from *Streptomyces* (Xia et al., 2014), algae-residue generated after lipid extraction from *Schizochytrium* sp. (Yin et al., 2019), wastewater from arachidonic acid and docosahexaenoic acid fermentation (Song et al., 2017), and both of wastewater and algae-residue from docosahexaenoic acid fermentation (Yin et al., 2018). These studies have achieved satisfying result by using fermentation waste. However, few studies focused on continuous recycling use of biomass residue and wastewater, which would be one of the most promising ways for raw material and waste treatment cost reduction in the long run.

In traditional fermentation industry, fermentation wastewater is discharged and biomass residue is abandoned after extraction for products, which would cause environmental pollution. In order to fully use these wastes, this study has tried to repeatedly use wastewater to replace fresh water. The yeast cells are hydrolyzed by enzymes and centrifuged to get lycopene product and biomass residue. Biomass residue is then used as nitrogen source to replace yeast extract. Furthermore, combination of wastewater and biomass residue is optimized for lycopene production. Based on the above study, a continuous recycling strategy using wastewater, biomass residue, and D-galactose is developed for lycopene production. This is the first time to report a continuous self-cycling fermentation system using wastewater, biomass residue and D-galactose to produce lycopene by *S. cerevisiae.* The comprehensive utilization of wastewater and biomass residue from lycopene production by *S. cerevisiae* and achievement of high lycopene titer will hopefully accelerate industrialization of microbial production of lycopene.

## Materials and Methods

### Microorganism

*Saccharomyces cerevisiae* SyBE_Sc14D14 was preserved in 25% (v/v) glycerol at -80°C in our lab (Chen et al., 2016).

### Medium and Culture Condition in Shake Flasks

Seed culture medium composition (weight/volume): 1% yeast extract, 2% tryptone, 2% glucose, uracil 0.005%. Fermentation medium composition (weight/volume): 3.0% yeast extract, 10% glucose, uracil 0.005%, D-galactose 1%.

For seed cultivation, three yeast single colonies were picked by a sterile shovel and transferred into a shake flask loaded with 20 mL seed culture medium. The seed shake flask was placed on a shaker with temperature set at 30°C and shaking speed 250 r/min overnight. Then the seed culture was inoculated into 1000 mL shake flask loaded with 200 mL seed culture medium and cultured for 8–10 h on a shaker with shaking speed at 250 r/min.

For shake flasks fermentation, 5 mL of the seed culture broth was transferred into a 250 mL shake flask loaded with 40 mL fermentation medium. The fermentation was performed on a shaker with temperature maintaining at 30°C and shaking speed at 300 r/min for 5 days.

### Fed-Batch Fermentation in 70 L Fermenter

For seed preparation, five of 1000 mL shake flasks loaded with 200 mL seed culture broth were combined to form 1000 mL of seed culture broth and then it was transferred into 70 L fermenter (Eastbio, China) loading with 40 L of seed culture medium. The temperature of seed cultivation was maintained at 30°C, aeration rate was set at 60 L/min, and the dissolved oxygen (DO) concentration was controlled above 20% by increasing agitation speed from 200 to 350 r/min while pH was not controlled.

When cell OD_600_ increased to 5∼6 in the seed fermenter, 4 L of the seed broth was piped to the 70 L fermenter loading with 40 L of fermentation medium. Fermentation temperature was maintained at 30°C, aeration rate was set at 80 L/min, pH was controlled between 5.8 and 6.4 by feeding 20% (weight/volume) sodium hydroxide solution, DO was controlled above 50% in the cell growth phase (0–40 h) and higher than 20% in the lycopene production phase (40 h∼ end) by adjusting agitation speed between 200 and 400 r/min.

During 0–40 h, 600 g/L glucose solution was continuously fed into the fermenter to maintain residual glucose concentration 0.5–1 g/L. For nitrogen source feeding, 600 g/L yeast extract solution was fed continuously into the fermenter. When cell growth slowed down (normally 40 h later), glucose and yeast extract feeding were stopped, then a final concentration of 20 g/L D-galactose in the fermentation broth was supplemented to activate lycopene biosynthesis. Then, ethanol was fed to the fermentation broth to maintain residual ethanol at 1–5 g/L by feeding 95% ethanol until the end of fermentation.

### Experiment Design

#### Wastewater and Biomass Residue Preparation

The fermentation broth was centrifuged at 4000 *g* for 10 min, after which the supernatant wastewater was gathered and stored at 4°C. Subsequently, the concentrated yeast cells were dissolved with tap water to OD_600_ of about 800 for cell lysis. The cells were disrupted by using a mixture of papain, lyticase, and glucanases (1%, w/v) which was provided by Angel Yeast Co., Ltd. The process was maintained at 55°C for 12 h. Next, the mixed solution was centrifuged at 4000 *g* for 10 min, the supernatant was lycopene product, and the biomass residue was harvested from the under layer which was stored at 4°C.

#### Optimization of Wastewater and Biomass Residue Replacement Ratio in Shake Flask Fermentation

##### Optimization of wastewater and biomass residue replacement

1. Optimization of wastewater replacement of fresh water: fresh water was replaced by 20, 40, 60, 80, and 100% wastewater, other medium components are 10% glucose, 1% D-galactose, 3% yeast extract, 0.005% uracil;
2. Optimization of D-galactose concentration in wastewater: 0, 0.2, 0.4, 0.6, 0.8, and 1% (w/v) D-galactose was added in the 100% wastewater, other medium components are 10% glucose, 3% yeast extract, 0.005% uracil;
3. Optimization of uracil concentration in wastewater: 0, 0.001, 0.002, 0.003, 0.004%, 0.005, and 0.01% uracil was added in the 100% wastewater, other medium components are 10% glucose, 3% yeast extract, 1% D-galactose;
4. Optimization of biomass residue replacement of yeast extract: 0.70 g/L KH_2_PO_4_ was added to the 3% yeast extract group, 3% yeast extract group without adding KH_2_PO_4_, yeast extract was replaced by 20, 40, 60, 80, and 100% biomass residue, other medium components are 100% fresh water, 10% glucose, 1% D-galactose, 0.005% uracil;
5. Optimization of different combinations of yeast extract and biomass residue: 0, 0.3, 0.6, and 0.9% (w/v) yeast extract was added in the 100% biomass residue group, other medium components are 100% wastewater, 10% glucose, 1% D-galactose, 0.004% uracil.

The experiments were performed in 250 mL shake flasks. Sampling was performed by taking three shake flasks for detection of lycopene titer and cell OD_600_.

##### Optimization of combination of wastewater and biomass residue

1. Traditional fermentation (Control): 100% fresh water combined with 3% yeast extract, other medium components are 10% glucose, 1% D-galactose, 0.005% uracil;
2. Wastewater combined with biomass residue (WW + BR): 100% wastewater combined with 8.4% biomass residue groups, other medium components are 10% glucose, 1% D-galactose, 0.004% uracil;
3. Wastewater combined with biomass residue supplementing yeast extract (WW + BR + YE): 100% wastewater combined with 8.4% biomass residue and 0.3% yeast extract, other medium components are 10% glucose, 1% D-galactose, 0.004% uracil;

The experiments were performed in 250 mL shake flasks. Sampling was performed every 12 h by taking three shake flasks for detection of lycopene titer and cell OD_600_.

##### Optimization of recycle times of wastewater, D-galactose, and biomass residue

1. Wastewater was recycled for 10 times: the medium components are 100% wastewater, 10% glucose, 3% yeast extract, 0.2% D-galactose, 0.004% uracil. In the sixth recycle, 0.5% D-galactose was additionally added in the medium.
2. Biomass residue was recycled for 10 times: the medium components are 100% fresh water, 10% glucose, 8.4% biomass residue, 0.3% yeast extract, 1% D-galactose, 0.004% uracil. In the sixth recycle, 0.4% yeast extract was additionally added in the medium.
3. Combination of wastewater and biomass residue was recycled for 10 times: the medium components are 100% wastewater, 10% glucose, 8.4% biomass residue, 0.4% yeast extract, 0.3% D-galactose, 0.004% uracil.

The experiments were performed in 250 mL shake flasks. Sampling was performed at the end of fermentation for detection of lycopene titer and cell OD_600_.

##### Fed-batch fermentation using wastewater and biomass residue in 70 L fermenter

The start batch was performed according to Section “Fed-Batch Fermentation in 70 L Fermenter.” At the end of fermentation, the fermentation broth was centrifuged by disk-type centrifuge at 6000 *g* continuously. The light fraction (wastewater) was piped out and was gathered in 100 L plastic drum while the heavy fraction (concentrated yeast cells) was transferred to a 50 L jacketed reactor and then a mixture of papain, lyticase, and glucanases (1% of the heavy fraction, w/v) was added to the reactor. The solution in the reactor was maintained at 55°C with agitation speed 100 r/min for 12 h. Next, the mixed solution was centrifuged at 4000 *g* for 10 min by a big bench centrifuge. The supernatant was lycopene product and the biomass residue was harvested from the under layer.

For fermentation recycle-1#, seed cultivation was performed the same as start batch. For the fermentation, the fresh water was replaced by wastewater gathered from the start batch. The total yeast extract used in the medium was decreased from 6.0 to 0.8% while the total D-galactose was decreased from 2.0 to 0.6%. Biomass residue was sterilized in a 20 L tank and piped into the fermenter the way yeast extract solution was fed in the start batch. Fermentation process parameters were set the same as the start batch. Reycle-2# and 3# were performed the same as recycle-1#.

#### Industrial Self-Cycling Process Design

Industrial self-cycling process was designed based on the result given by 70 L fermenters. At the end of fermentation, the fermentation broth was centrifuged by an industrial disk-type centrifuge. The supernatant was wastewater which was pumped to the sterilization system to substitute fresh water. The under layer wet biomass was transferred to another tank with agitation speed 80 r/min for enzymatic hydrolysis in which the same concentration of enzymes, temperature, and time were performed. Then, the enzymatic hydrolysis liquid was centrifuged by the same industrial disk-type centrifuge. The supernatant lycopene crystal was collected and the under layer was biomass residue which was piped to the sterilization system for sterilization. Then it was stored in a sterilized tank as nitrogen source feeding for lycopene production. In this recycling system, certain amount of fresh water was used to replenish the loss of water, D-galactose and yeast extract were designed to be additionally supplemented to maintain cell growth and lycopene production according to the optimal process developed in 70 L fermenter.

### Analytical Methods

#### Assay of Glucose, Ethanol, Total Nitrogen, Phosphorus, Amino Acids, and D-Galactose

Glucose and ethanol were monitored by glucose/ethanol enzyme membrane reaction analyzer (SBA-40C, Shandong Academy of Sciences). Total nitrogen was measured by Kjeldahl determination based on ISO 8968-1 (2014). Total phosphorus was measured by ammonium molybdate spectrophotometry determination based on ISO 6491 (1998). Amino acid was measured according to Chen et al. (2008). For the rapid assay of D-galactose, lactose/galactose (rapid) assay kit (Megazyme) was used.

#### Extraction and Analysis of Lycopene

0.1 mL fermentation broth was accurately measured and added into a 2 mL Eppendorf tube containing 1 mL tetrahydrofuran, then a mixture of 3.2 and 1.2 mm zirconia beads was added to the mixed solution. The Eppendorf tube was placed in the Automatic sample rapid grinding machine (Jing Xin, JXFSTPRP-24) with vibration speed 70 Hz for 5 min. After centrifugation (9690 *g* for 2 min), the supernatant was transferred to a 10 mL volumetric bottle with a pipette. Then 1 mL fresh tetrahydrofuran was added into the residue in the Eppendorf tube for the second round of extraction. The extraction was repeated at least five times until no red color was observed in the residue. All the tetrahydrofuran using for lycopene extraction was gathered and transferred to the 10 mL volumetric bottle. Finally, fresh tetrahydrofuran was added to the 10 mL volumetric bottle to get a final volume of 10 mL lycopene extraction solution. The lycopene extraction solution was then filtered by 0.2 μm filter for further analysis. The lycopene content was analyzed by an HPLC system (Waters e2695) equipped with BDS Hypersil—C18 column (4.6 × 150 mm, 5 μm). UV/VIS detector was set at 471 nm. Mobile phase was consisted of methanol/acetonitrile/dichloromethane with the volume ratio 42/42/16 and the flow rate was 1 mL/min. The temperature of column system was set at 30°C during the whole analyzing process.

#### Statistical Analysis

Analysis of independent-samples *T*-test was performed using the software SPSS 17.0 (IBM, United States).

## Results and Discussion

### Composition Analysis of Yeast Extract, Biomass Residue, and Wastewater

In order to reuse the waste generated from lycopene production, composition of wastewater and biomass residue was analyzed and compared with yeast extract. Since yeast cells could be broken down by proteases (Verduyn et al., 1999; Chae et al., 2001), a mixture of papain, lyticase, and glucanases is used in this study to break down yeast cells. When the cells are broken down, lycopene crystal is released from the cell membrane. After centrifugation, biomass residue is harvested from the under layer. The components of yeast extract, wastewater, and biomass residue are shown in Table 1.

**TABLE 1:** Analysis of nutrients in yeast extract, biomass residue, and wastewater.

**Nutrients**	**Yeast extract**	**Biomass residue**	**Wastewater**
D-galactose	0	1.1 (g/kg)	6.6 (g/kg)
Total phosphorus	0.0096 (g/g DCW)	0.0044 (g/g DCW)	35 (mg/100 mL)
Total nitrogen	0.0995 (g/g DCW)	0.0460 (g/g DCW)	12 (mg/L)

	**Content (% of dry matter)**		
**Amino acid**	**Yeast extract**	**Biomass residue**	

Aspartic acid	6.58	2.71	
Threonine	2.91	1.63	
Serine	2.95	1.63	
Glutamate	11.78	5.97	
Glycine	2.72	2.53	
Alanine	7.04	4.99	
Cysteine	1.15	0.16	
Valine	3.48	1.27	
Methionine	0.88	0.07	
Isoleucine	2.97	0.43	
Leucine	4.31	0.29	
Tyrosine	2.12	0.38	
Phenylalanine	1.84	2.26	
Lysine	4.52	0.72	
Histidine	1.20	0.35	
Arginine	3.51	0.84	
Proline	2.26	2.53	

As shown in Table 1, D-galactose remains a concentration of 6.6 g/L in wastewater and 1.1 g/L in biomass residue and the total loss of D-galactose is 45.8%. Obviously, D-galactose is not used up because the genes involved in metabolism of D-galactose were knocked out in the strain used in this study (Chen et al., 2016). As a result, D-galactose is merely used as an inducer. Since D-galactose is mostly left in wastewater, it may be repeatedly used for lycopene production accompanied by wastewater recycling use. Table 1 also shows that the total phosphorus and nitrogen in biomass residue are 54.17 and 53.77% lower than yeast extract, respectively. In addition, the contents of amino acid are different in biomass residue and yeast extract. Yeast extract is a widely used multi-functional nitrogen source in microbial fermentation industry (Li et al., 2018). Though the content of nitrogen and phosphorus in biomass residue are lower than in yeast extract, the species of amino acid shows no difference in both the two nitrogen sources. By increasing concentration of biomass residue or supplementing additional amount of yeast extract, the lacked N and P elements in biomass residue could be increased to meet the need of cell growth. Using this strategy, it is hopefully that biomass residue could be used as nitrogen source for *S. cerevisiae* to produce lycopene.

### Effect of Wastewater and Biomass Residue Replacement on Lycopene Production

Effects of different wastewater replacement (0, 20, 40, 60, 80, and 100%) of fresh water on cell growth and lycopene production were studied. Glucose, yeast extract, D-galactose, and uracil were added the same as traditional fermentation. Further on, considering that abundant D-galactose is existed in wastewater, the effects of different concentration of D-galactose and uracil combining with the optimal wastewater replacement on cell growth and lycopene production were also studied. Yeast extract is a widely used nitrogen source not only for microbial growth but also showed significant effect on useful metabolites production (Lo et al., 1997; Bury et al., 2018). Biomass residue used as nitrogen source to replace yeast extract was also optimized in this study.

**FIGURE 1:**
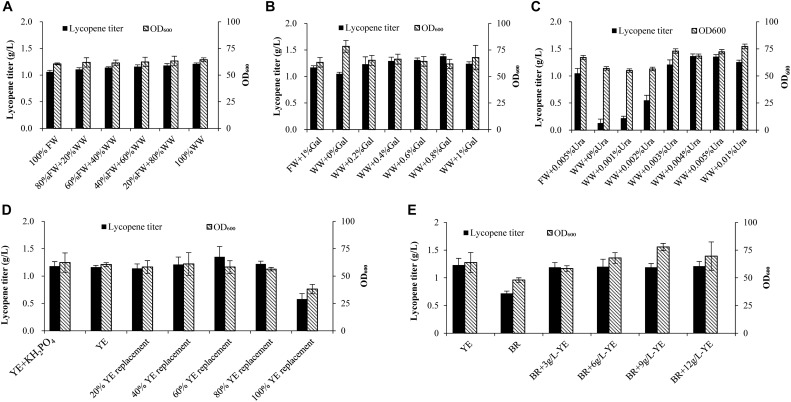
Effects of wastewater and biomass residue replacement of fresh water and yeast extract on cell growth and lycopene production. **(A)** Fresh water was replaced by 20, 40, 60, 80, and 100% wastewater, other medium components are 10% glucose, 1% D-galactose, 3% yeast extract, 0.005% uracil. **(B)** 1% D-galactose was added in the 100% fresh water, 0, 0.2, 0.4, 0.6, 0.8, 1% D-galactose was added in the 100% wastewater, other medium components are 10% glucose, 3% yeast extract, 0.005% uracil. **(C)** 0.005% Uracil was added in the 100% fresh water, 0, 0.001, 0.002, 0.003, 0.004, 0.005, 0.01% uracil was added in the 100% wastewater, other medium components are 10% glucose, 3% yeast extract, 1% D-galactose. **(D)** 0.70 g/L KH_2_PO_4_ was added to the 3% yeast extract group, 3% yeast extract group without KH_2_PO_4_, yeast extract was replaced by 20, 40, 60, 80, and 100% biomass residue, other medium components are 100% fresh water, 10% glucose, 1% D-galactose, 0.005% uracil. **(E)** Nitrogen source was provided by different combinations of yeast extract and biomass residue, other medium components are 100% fresh water, 10% glucose, 1% D-galactose, 0.005% uracil. Experiments were performed in shake flask fermentation. Results are mean ± SD of three determinations.

As seen from Figure 1A, wastewater replacement of fresh water varied from 20 to 100% shows no significant effect on cell growth. However, when fresh water is totally replaced by wastewater, lycopene titer attains 1.21 g/L, which is 14.2% higher than the fresh water group. Some studies find that cell growth would decline when they started to produce toxic compounds (Cartenì et al., 2012; Mazzoleni et al., 2015). The self-produced inhibitors would be a limitation for high cell density cultivation (Mazzoleni et al., 2015). Oppositely in our study, wastewater shows no adverse effect on cell growth. The possible reason was that the fermentation was ended before the cells stepped into decline phase. As a result, the concentration of inhibitors may not be sufficient enough to exert adverse effect on the cell growth. Surprisingly, the lycopene titer increases by using wastewater to substitute fresh water. It is inferred that some of intermediates generated in the tricarboxylic acid cycle or mevalonic acid pathway may be left in wastewater. These intermediates are important precursors for lycopene production (Bhosale, 2004) and they may be channeled into lycopene synthesis pathway as wastewater is recycled.

Further on, different concentration of D-galactose (0, 0.2, 0.4, 0.6, 0.8%) combined with 100% wastewater were applied in the shake flask fermentation. Figure 1B shows that when wastewater is applied to the lycopene production without adding D-galactose, lycopene titer (1.05 g/L) is slightly lower than the control (1.17 g/L). While adding 2, 4, 6, 8, 10 g/L D-galactose, the lycopene titers reach 1.23, 1.29, 1.31, 1.38, 1.24 g/L, respectively. The result clearly shows that the residual D-galactose can be reused for activating lycopene synthesis.

The strain used in this study is uracil defective type. Thus, uracil is imperative for cell growth. In order to find out the optimal amount of uracil needed when wastewater is used, different concentrations of uracil combined with 100% wastewater are prepared for lycopene production in shake flask fermentation. Figure 1C shows that cell OD_600_ and lycopene titers are significantly lower than the control when the uracil concentration is lower than 0.002%. As the uracil concentration increases to 0.003%, cell OD_600_ attains 72.96 while lycopene titer reached 1.21 g/L, which are 8.90 and 15.24% higher than the control, respectively. The highest lycopene titer of 1.37 g/L is achieved when adding 0.004% uracil, which is 30.13% higher than the control. So the optimal concentration of uracil is 0.004%.

A total nitrogen source of 3% (weight/volume) yeast extract was used in the control experiments, followed by were 20, 40, 60, 80, 100% replacement ratios of yeast extract with biomass residue. The exact demand of total nitrogen is calculated based on 3% yeast extract. Since the phosphorus in 100% replacement group is 60.2% higher than the yeast extract group. Another control group is added 0.70 g/L KH_2_PO_4_ to keep the same phosphorus level as the 100% replacement group (Supplementary Table S1). Glucose, D-galactose, and uracil are added the same as the control group. As shown in Figure 1D, no significant differences of lycopene titer and cell OD_600_ are observed when additional KH_2_PO_4_ is added in the yeast extract group, meaning that phosphorus is not the limited nutrition in the current fermentation system. It is clearly showed that when replacement ratios of yeast extract by biomass residue were 20, 40, 60, and 80%, the lycopene titers and cell OD_600_ show no significant difference. The mean titers and cell OD_600_ are 1.23 g/L and 58.45, while the yeast extract group is 1.16 g/L and 60.49, respectively. In contrast, the lycopene titers and cell OD_600_ decrease significantly as the replacement ratio increases to 100%. Glucose consumption rate in the 100% replacement group significantly reduces compared to the yeast extract group. Since glucose is the main carbon source in cell growth, the glucose consumption rate could reflect the primary metabolic situation (Zaman et al., 2009). So, the decreased glucose consumption rate may be caused by two reasons: one is that toxic metabolic compounds are generated in cells, which affect the cell growth (Cartenì et al., 2012; Mazzoleni et al., 2015). The other reason is that biomass residue lacks some components which play key roles in cell growth and lycopene production. However, as previously discussed, wastewater is proved to contain no toxic compounds that would affect cell growth. Thus, it is speculated that 100% replacement group lacks some components which can be found in yeast extract like microelement, vitamins, and nucleotides, which are key important for cell growth and products synthesis (Shamzi et al., 2013; Lee et al., 2014). Based on the above speculation, 3, 6, 9, 12 g/L yeast extract were supplemented to the 100% replacement group (BR). Figure 1E shows that cell OD_600_ and lycopene titer increase as yeast extract increases from 3 to 12 g/L with the lycopene titer attaining 1.21 g/L, which is 77.9% higher than the biomass residue group. Cell OD_600_ in BR + 9 g/L YE group reaches 77.8, which was 24.88% higher than the BR group. Time courses of glucose consumption rate, lycopene production, and cell OD_600_ in BR + 3 g/L YE groups are similar to the YE group (Supplementary Material), which means that addition of 3 g/L yeast extract in the biomass residue group could sufficiently provide microelement, vitamins, and nucleotides which may not exist in biomass residue. Yeast extract is identified as significant factor for producing many valuable nutrition like arachidonic acid, docosahexaenoic acid, astaxanthin, and beta-carotene (Kusdiyantini et al., 1998; Bhosale and Gadre, 2001; Quilodrán et al., 2010; Li et al., 2018). The same phenomenon is also observed in this study. This proves that the lycopene production system is very dependent on yeast extract. Thus, the recycling use of biomass residue should also be supplemented at least 3 g/L yeast extract to maintain normal yield of lycopene.

According to the above results, wastewater could completely replace fresh water in the fermentation medium for lycopene production while only 0.2% D-galactose and 0.003% uracil are needed to maintain the lycopene titer. In addition, biomass residue combined with 3 g/L yeast extract could completely replace 30 g/L yeast extract while lycopene titer is not significantly decreased than the YE group. The recycling use of wastewater and biomass residue could greatly lower the cost of raw materials as well as the waste treatment and environmental pollution from lycopene production.

### Effect of Combination of Wastewater and Biomass Residue on Lycopene Production

**FIGURE 2:**
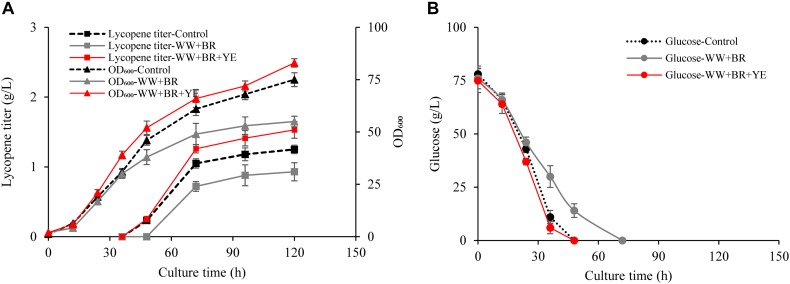
Effects of combination of biomass residue and wastewater on lycopene production. **(A)** Time courses of cell growth and lycopene titer for 100% fresh water combined with 3% yeast extract groups (Control), 100% wastewater combined with 8.4% biomass residue groups (WW + BR), 100% wastewater combined with 8.4% biomass residue and 0.3% yeast extract groups (WW + BR + YE). **(B)** Time courses of glucose consumption for Control, WW + BR, and WW + BR + YE groups. Experiments were performed in shake flask fermentation. Data are mean ± SD of three determinations.

Based on the optimal fermentation condition developed in Section “Effect of Wastewater and Biomass Residue Replacement on Lycopene Production,” combination of wastewater and biomass residue with additional yeast extract supplementation (WW + BR + YE) was designed as a novel fermentation medium for lycopene production. In addition, wastewater combined with biomass residue without supplementing any yeast extract (WW + BR) was also used for lycopene production. The results are shown in Figure 2.

Obviously, lycopene titer in the WW + BR group is much lower than the control (Figure 2A) and the glucose consumption rate is significantly lower in the WW + BR group than in the control group (Figure 2B). At the end of fermentation, the lycopene titer of WW + BR group is 0.93 g/L, which is 25.6% lower than the control group but 29.2% higher than the FW + BR group, which verifies the hypothesis that biomass residue lacked some key growth factors needed for lycopene synthesis, because some of the lacked factors go to the wastewater while some are metabolized by the cells. This is in coincidence with the finding of Yin et al. (2019) that 20% wastewater combined with algae residue could enhance docosahexaenoic acid production. However, the combination of wastewater and biomass residue lowers lycopene production and cell growth significantly than the control, which means that the impact of nitrogen source on lycopene production is more significant than wastewater. While feeding 3 g/L yeast extract to the WW + BR group, lycopene titer reaches 1.53 g/L and cell OD_600_ reaches 83, which are 22.4 and 10.7% higher than the control (*P* < *0.05*), respectively.

The results of this experiment shows that combination of 100% wastewater, 0.2% D-galactose, 8.4% biomass residue, and 0.3% yeast extract could replace the traditional fermentation medium without any adverse effect on cell growth and lycopene production. The recycling use of both the wastewater and biomass residue could significantly lower the cost of fermentation raw materials.

### Effect of Recycle Times of Wastewater, D-Galactose, Biomass Residue, and Their Combination on Lycopene Production

**FIGURE 3:**
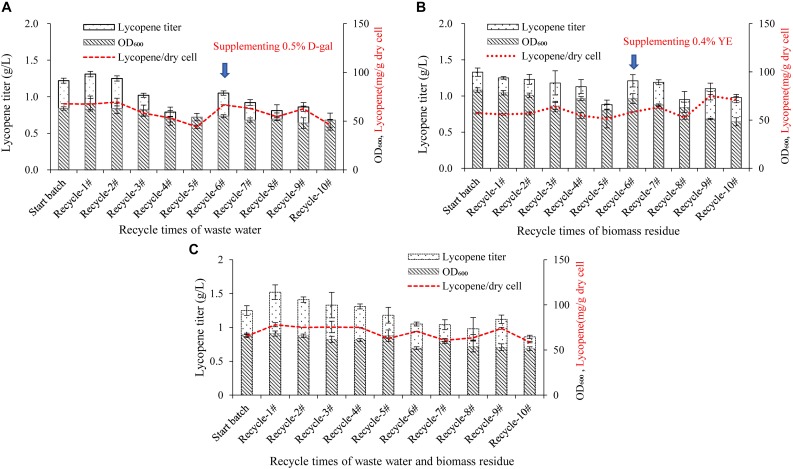
Effect of recycle times of wastewater, D-galactose, biomass residue, and their combination on cell growth and lycopene production. **(A)** Wastewater was recycled for 10 times. The medium components are 100% wastewater, 10% glucose, 3% yeast extract, 0.2% D-galactose, 0.004% uracil. In the sixth recycle, 0.5% D-galactose was additionally added in the medium. **(B)** Biomass residue was recycled for 10 times. The medium components are 100% fresh water, 10% glucose, 8.4% biomass residue, 0.3% yeast extract, 1% D-galactose, 0.005% uracil. In the sixth recycle, 0.4% yeast extract was additionally added in the medium. **(C)** Combination of wastewater and biomass residue was recycled for 10 times. The medium components are 100% wastewater, 10% glucose, 8.4% biomass residue, 0.4% yeast extract, 0.3% D-galactose, 0.004% uracil. Experiments were performed in shake flask fermentation. Data are mean ± SD of three determinations.

The recycle times of wastewater and biomass residue would be key important for an economical production system because the more recycle times performs, the more cost would be reduced and the less waste would be discharged. As shown in Figure 3A, the lycopene titers in the recycle-1# and 2# are equivalent to the start batch, but decreases from the recycle-3#. In the recycle-5#, the lycopene titer is significantly lower than the start batch while cell OD_600_ is also lower. It is inferred that in the first two recycles, the toxic compounds generated by cells are not sufficient to affect the cell growth and lycopene production. However, as the recycle times increases, the accumulation of toxic compounds starts to play adverse role (Cartenì et al., 2012; Mazzoleni et al., 2015). In the study by Song et al. (2017), wastewater could only be recycled for one time due to the accumulated inhibitors. This might be due to the different strain used in the study as well as the duration of fermentation. In the recycle-6#, 5 g/L D-galactose is supplemented in the fermentation system additionally, the lycopene titer is increased to 1.05 g/L which is 54.4% higher than the recycle-5# but is still 13.93% lower than the start batch while cell OD_600_ is 12.61% lower (*P* < *0.05*). This meant that lack of D-galactose would also lower lycopene synthesis. However, after supplementing enough D-galactose, cell OD_600_ and lycopene titer still shows the same trend when the recycle keeps going on. Oppositely, the recycling of biomass residue shows more stable than the wastewater recycle (Figure 3B). The lycopene titer and cell growth are not significantly lowered until the recycle-5#. In the recycle-6#, additional 3 g/L yeast extract is supplemented in the fermentation system and the lycopene titer regains 1.21 g/L. However, in the recycle-8#, lycopene titer and cell growth decreases again.

Further on, the effect of recycle times of wastewater, D-galactose, biomass residue combination on cell growth, and lycopene production was studied. The medium is consisted of 100% wastewater, 10% glucose, 8.4% biomass residue, 0.4% yeast extract, 0.3% D-galactose, and 0.004% uracil. The result is presented in Figure 3C. The lycopene production is stable in the first five recycles. However, in the sixth recycle, the lycopene titer decreases 16% comparing with the start batch. The result indicates that recycling use of wastewater and biomass residue could last for five times while lycopene titer is not significantly decreased.

### Fed-Batch Fermentation Using Wastewater and Biomass Residue in 70 L Fermenter

**FIGURE 4:**
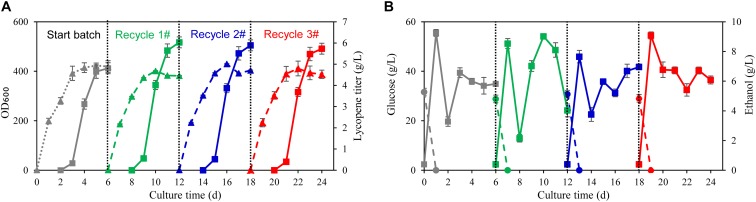
Fed-batch fermentation using wastewater and biomass residue in 70 L fermenter. **(A)** Time courses of cell growth and lycopene titer in the traditional fermentation process by using fresh water and yeast extract (start batch) and the self-cycling fermentation process by using wastewater and biomass residue (recycle 1, 2, 3#). The solid triangle symbols represent cell OD_600_ and the solid squares represent the lycopene titer. **(B)** Time courses of residual glucose and ethanol in the traditional fermentation process by using fresh water and yeast extract (start batch) and the self-cycling fermentation process by using wastewater and biomass residue (recycle 1, 2, 3#). The solid circle symbols represent ethanol concentration in the fermentation broth and the solid squares represent the residual glucose concentration in the fermentation broth. Experiments were performed in 70 L fermenter. Data are mean ± SD of three determinations.

To verify the recycling strategy of wastewater and biomass residue developed in shake flasks fermentation, fed batch fermentation using wastewater and biomass residue was carried out in 70 L fermenters. The glucose used in the traditional fermentation process was 200 g/L and yeast extract was 60 g/L. By such strong feeding of carbon and nitrogen source, the highest cell OD_600_ reaches 415 at 91.4 h while the lycopene titer reaches 4.81 g/L (Figure 4A). The fermentation process using self-cycling strategy shows almost the same consumption rates of glucose and ethanol as the traditional fermentation but only 8 g/L yeast extract was used (Figure 4B). Attractively, the lycopene titer attains as high as 6.03 g/L which is 25.4% higher than the traditional process (*P* < 0.05), showing the same trend as in shake flasks fermentation (Figure 4A). In the following recycle 2 and 3#, lycopene titer reaches 5.89 and 5.73 g/L, respectively.

The total nitrogen (N) and phosphorus (P) balance in the self-cycling fermentation process is calculated and presented in Table 2. In the start batch, the total N in the medium is 170.74 g while the output of total N is 166.69 g, which is very close. The total P in the start batch is 16.47 g while the output of total P is 15.26 g which is also well matched. However, in the recycle-1#, the total N and P increases due to the additional supplement of yeast extract, but the total N and P also matches between the input and output. In a word, the N and P in the whole process showed balance between the input and output, which is a good basis for designing a continuous self-cycling fermentation process using waste byproducts generated in the lycopene production.

**TABLE 2:** Nitrogen and phosphorus balance in the self-cycling fermentation process.

***Medium input-start batch***				***Products out-start batch***			
	
**IN**	**Weight (g)**	**Total N(g)**	**Total P (g)**	**OUT**	**Weight (g)**	**Total N(g)**	**Total P (g)**
Yeast extract	2280	158.80	15.32	Biomass residue	6232	155.57	14.88
Fresh water	40000	0.00	0.00	Wastewater	31768	11.12	0.38
Seed inoculum	120	11.94	1.15	Lycopene product	425	\	\
Total		170.74	16.47	Total		166.69	15.26

***Medium input-recycle #1***				***Products out-recycle #1***			
	
**IN**	**Weight (g)**	**Total N(g)**	**Total P (g)**	**OUT**	**Weight (g)**	**Total N(g)**	**Total P (g)**

Biomass residue	6232	155.57	14.88	Biomass residue	6816	176.76	16.76
Yeast extract	296.4	20.64	1.99	Wastewater	33000	20.79	0.63
Wastewater	31768	11.12	0.38	Lycopene product	457	\	\
Seed inoculum	120	11.94	1.15				
Total		199.28	18.41	Total		197.55	17.39

### Design of a Continuous Self-Cycling Fermentation System

**FIGURE 5:**
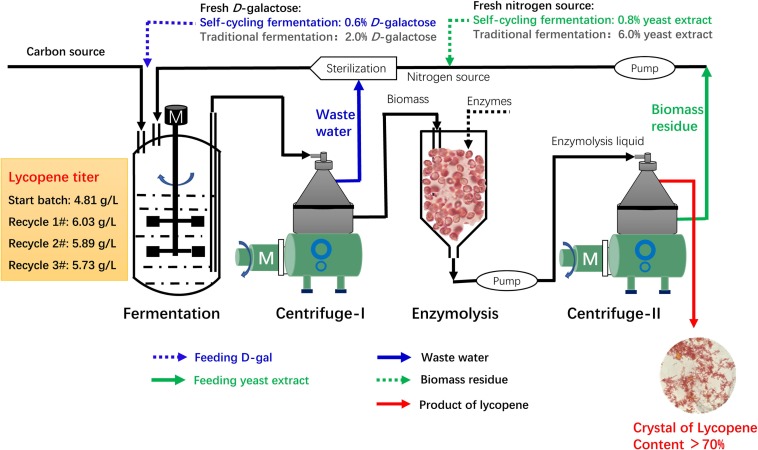
Overall design of a continuous self-cycling fermentation system for industrial production of lycopene.

Based on the above results, a continuous self-cycling fermentation system using biomass residue, wastewater, and D-galactose is designed as shown in Figure 5. At the end of fermentation, the fermentation broth is centrifuged in an industrial centrifuge. The supernatant wastewater is pumped to the sterilization system to substitute fresh water. The under layer wet biomass is transferred to another tank for enzymatic hydrolysis. The enzymatic hydrolysis liquid is then centrifuged. The supernatant lycopene crystal is collected and the under layer biomass residue is used as nitrogen source for lycopene production.

**FIGURE 6:**
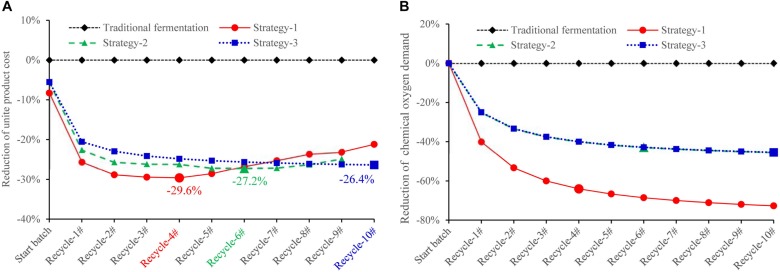
Comparison of unite lycopene production cost **(A)** and unite chemical oxygen demand **(B)** using different strategies. The unite cost = accumulative total cost/accumulative total lycopene output in traditional fermentation (rhombus symbols): Fermentation medium composition: 100% fresh water, 2% D-galactose, 6% yeast extract, 0.005% uracil, 12% ethanol, 20% glucose; self-cycling strategy-1 (circle symbols): Fermentation medium composition: 70% wastewater, 30% fresh water, 0.6% D-galactose, 0.8% yeast extract, 0.004% uracil, 12% ethanol, 20% glucose; self-cycling strategy-2 (triangle symbols): Fermentation medium composition: 50% wastewater, 50% fresh water, 1% D-galactose, 1.2% yeast extract, 0.004% uracil, 12% ethanol, 20% glucose; self-cycling strategy-3 (square symbols): Fermentation medium components: 50% wastewater, 50% fresh water, 1.2% D-galactose, 3.0% yeast extract, 0.004% uracil, 12% ethanol, 20% glucose.

In this recycling system, certain amount of fresh water, D-galactose, and yeast extract is designed to be additionally supplemented to maintain cell growth and lycopene production. According to our results, an assumption can be made that when concentration of biomass residue and wastewater decreases, yeast extract increases, the recycle times of wastewater and biomass residue would be increased without lowering lycopene titer. The self-cycling strategy 1 is based on lab experiments, a total of 30% fresh water, 8 g/L yeast extract, 6 g/L D-galactose is additionally supplemented to the system. The self-cycling strategy 2 uses 50% fresh water, 12 g/L yeast extract, 10 g/L D-galactose. The self-cycling strategy 3 uses 50% fresh water, 30 g/L yeast extract, 12 g/L D-galactose. Raw material cost containing enzymes for hydrolysis of biomass residue decreases -61.1, -48.0, -33.2% in the strategy 1, 2, and 3 (the details for the cost of each item could be found in Supplementary Table S3). D-galactose accounts for 52.8% in the raw material which is very high. However, by recycling use of wastewater, the cost of D-galactose is cut down by 70.0%. Except for this strategy, choosing a cheap inducer by construction of a new strain can be tried so as to greatly lower the cost from the beginning. Furthermore, in the optimization of fermentation medium, yeast extract should be considered to be replaced by inorganic nitrogen source such as ammonia to greatly reduce the cost of raw materials. However, it should be noted that inorganic nitrogen contains no other component than pure N element, which may present a challenge to its application in lycopene production. Essential amino acids, minerals, trace elements, etc., should be provided for cell growth and lycopene production. These are two targets which could be set in our future work. Figure 6A shows the reduction of unite product cost by using different strategies. The lowest unite cost shows in the strategy 1 and 2 are recycle-4# and 6#, respectively. It also shows that the cost of strategy 3 decreases from recycle-1# to recycle-10#, though the trend becomes flat. When we focus on the unite product cost, the strategy 3 would be the optimal one because it could run more times without increasing the unite product cost. However, the strategy 2 and 3 shows less chemical oxygen demand reduction since 50% wastewater is discharged (Figure 6B). Oppositely, chemical oxygen demand in the strategy 1 continuously decreased with the recycle running. At the recycle-4#, chemical oxygen demand decreased 64.0% compared to traditional fermentation. In conclusion, applying this continuous self-providing fermentation strategy, the raw material cost and unite product cost significantly decreases and waste discharge is also greatly reduced. The industrial recycling model may be well used in the future.

## Conclusion

This study uses wastewater and biomass residue as cost-free raw materials for lycopene production in *S. cerevisiae*. A continuous recycle strategy using wastewater, biomass residue, and D-galactose is developed for lycopene production, whereby the mean lycopene titer of the first five recycles attains 1.33 ± 0.123 g/L. Finally, the strategy is applied in 70 L fermenter with lycopene titer attaining 5.88 ± 0.15 g/L in three recycles, which is 22.25% higher than the start batch (*P* < 0.05). This study provides a novel method for producing lycopene economically and decreasing waste significantly, which also shows potential use for other industrial production of microbial products by *S. cerevisiae*.

## Data Availability Statement

All datasets generated for this study are included in the article/Supplementary Material.

## Author Contributions

ZW wrote the manuscript with the assistance of all other authors and contributed to conceptualization, methodology, investigation, and writing. XL contributed to investigation, data curation, and writing—original draft preparation. CY contributed to investigation, visualization, and writing—original draft preparation. SL contributed to project administration, supervision, and validation. SX contributed to investigation. YY contributed to funding acquisition, writing—reviewing and supervision.

## Conflict of Interest

The authors ZW, XL, CY, SL, and SX are employed by the company CABIO Biotechnology (Wuhan) Co., Ltd. The remaining author declares that the research was conducted in the absence of any commercial or financial relationships that could be construed as a potential conflict of interest.
