# Supplementary material for: Continuous Self-Cycling Fermentation Leads to Economical Lycopene Production by Saccharomyces cerevisiae
Source: Front Bioeng Biotechnol. 2020 May 15;8:420. doi: 10.3389/fbioe.2020.00420 (PMC7242880; doi:10.3389/fbioe.2020.00420)
Supplement: Supplementary file 1 [file Data_Sheet_1.docx]

**Supplementary material**


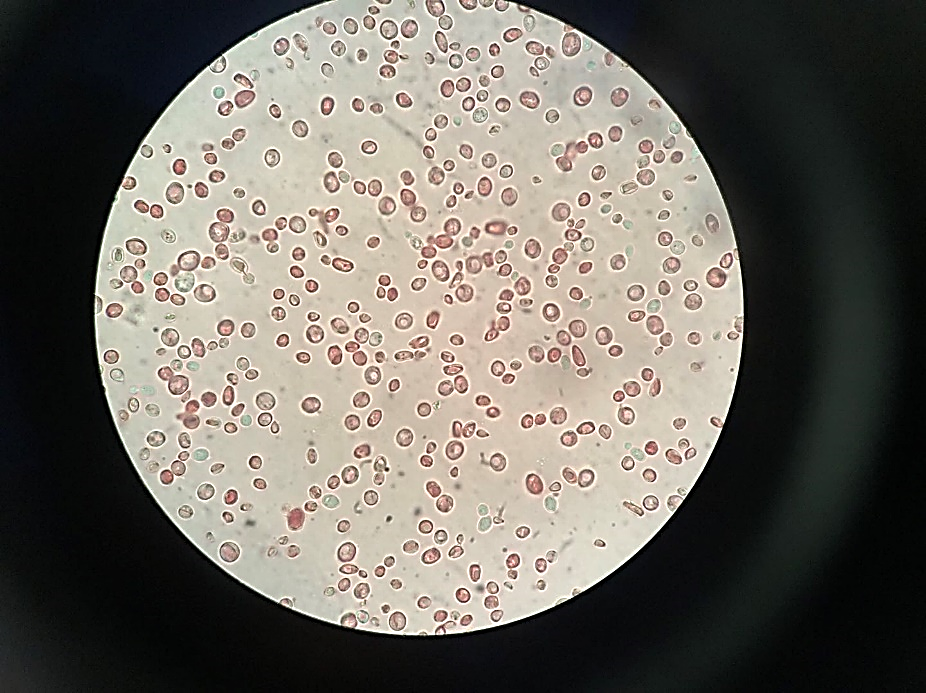
**
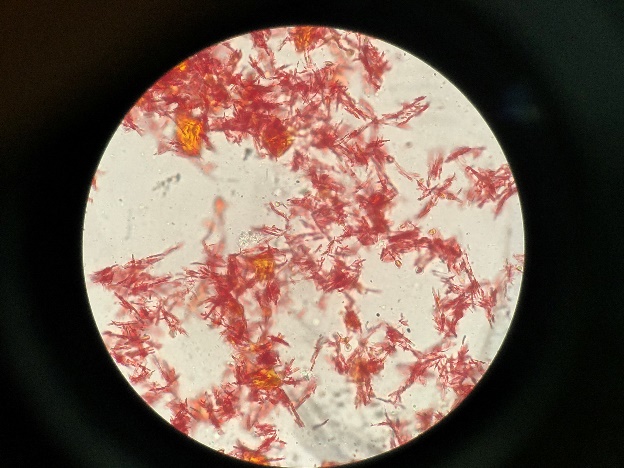
**

**(B)**

**(A)**

Supplementary Figure 1. Morphologies of cells and lycopene crystal under the microscope with 1000 times magnification. (A) Fermentation sample was taken at 120 h. (B) Sample were taken from the upper layer of centrifugation of enzymatic liquid.


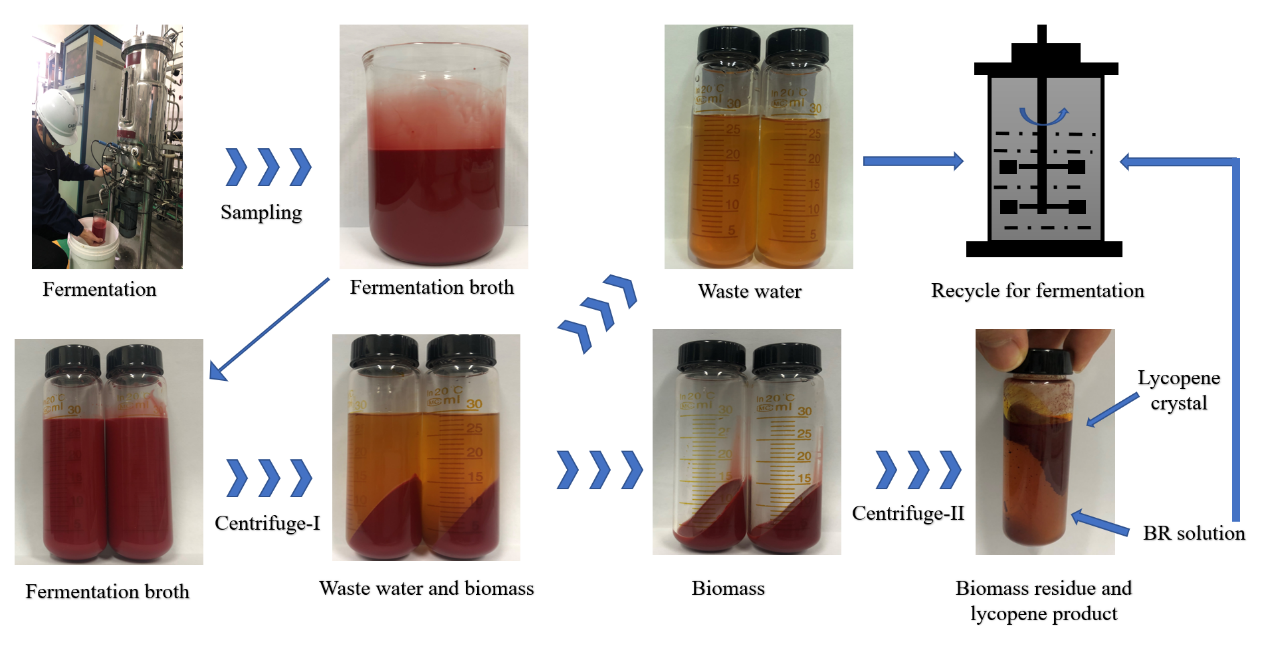


Supplementary Figure 2. Overall design of recycle use of waste water and biomass residue in the 70 L fermenter.

Supplementary Figure 3. Effect of biomass residue used as nitrogen source on lycopene production and cell growth in shake flask fermentation. (A) Time courses of cell growth, lycopene titer and residual glucose for yeast extract and 100% biomass residue groups. Yeast extract was replaced by 100% biomass residue, other medium composition was 100% fresh water, 10% glucose, 1% *D*-galactose, 0.005% uracil. (B) Time courses of cell growth, lycopene titer and residual glucose for yeast extract and 100% biomass residue combined with 3 g/L yeast extract groups. Nitrogen source was provided by yeast extract and biomass residue, other medium composition was 100% fresh water, 10% glucose, 1% *D*-galactose, 0.005% uracil. Experiments were performed in shake-flask fermentation. Data were mean ± SD of three determinations.

**Table S1 Amount and distribution of total nitrogen, phosphorus in fermentation medium for different replacement ratio of yeast extract by biomass residue**

| Replacement ratio of yeast extract by biomass residue | Amount of nitrogen source used in fermentation (g/L) | | Total N of the medium (g/L) | Total P of the medium | Distribution of total N (g/L) | | Distribution of total P (g/L) | |
| --- | --- | --- | --- | --- | --- | --- | --- | --- |
|  | Biomass residue | Yeast extract | Biomass residue+yeast extract | Biomass residue+yeast extract | Biomass residue | Yeast extract | Biomass residue | Yeast extract |
| 0%+KH_2_PO_4_ | 0 | 30 | 2.1 | 0.37 |  |  | 0 | 0.37 |
| 0% | 0 | 30 | 2.1 | 0.29 | 0.0 | 2.1 | 0.00 | 0.21 |
| 20% | 17 | 24 | 2.1 | 0.30 | 0.4 | 1.7 | 0.07 | 0.17 |
| 40% | 34 | 18 | 2.1 | 0.32 | 0.8 | 1.3 | 0.13 | 0.13 |
| 60% | 50 | 12 | 2.1 | 0.34 | 1.3 | 0.8 | 0.20 | 0.08 |
| 80% | 67 | 6 | 2.1 | 0.35 | 1.7 | 0.4 | 0.27 | 0.04 |
| 100% | 84 | 0 | 2.1 | 0.37 | 2.1 | 0.0 | 0.34 | 0.00 |

**Table S2 Parameters of medium input and products in 70 L fermenter in the start batch and recycle batch #1**

| **Medium input-start batch** |  |  |  | **Products out- start batch** |  |  |  |
| --- | --- | --- | --- | --- | --- | --- | --- |
| Fresh water volume |  | 40000 | mL | Fermentation broth volume |  | 38000 | mL |
| Dry biomass |  | \ |  | Dry biomass |  | 89 | g/L |
| Wet biomass/Volume |  | \ |  | Wet biomass/Volume |  | 16.40% | v/v |
| Yeast extract | Weight | 2280 | g | Biomass residue | Weight | 6232 | g |
|  | Dry biomass | 70.0% | g/g |  | Dry biomass | 54.3% | g/g |
|  | Total N content | 9.95% | g/g |  | Total N content | 4.60% | g/g |
|  | Total P content | 0.96% | g/g |  | Total P content | 0.44% | g/g |
| Seed inoculum | Volume | 4000 | mL | Waste water | Volume | 31768 | mL |
|  | Total N content | 9.95% | g/g |  | Total N content | 35 | mg/100ml |
|  | Total P content | 0.96% | g/g |  | Total P content | 12 | mg/l |
| **Medium input -recycle #1** |  |  |  | **Products out -recycle #1** |  |  |  |
| Waste water |  | 31770 | mL | Fermentation broth volume |  | 39400 | mL |
| Fresh water |  | 8230 | mL | Dry biomass |  | 92.50 | g/L |
| Wet biomass/Volume |  | 16.40% | v/v | Wet biomass/Volume |  | 17.30% | v/v |
| Yeast extract | Weight | 296.4 | g | Biomass residue | Weight | 6816.2 | g |
|  | Dry biomass | 70.0% | g/g |  | Dry biomass | 53.5% | g/g |
|  | Total N content | 9.95% | g/g |  | Total N content | 4.85% | g/g |
|  | Total P content | 0.96% | g/g |  | Total P content | 0.46% | g/g |
| Seed inoculum | Volume | 4000 | mL | Waste water | Volume | 32583.8 | mL |
|  | Total N content | 120 | mg/100ml |  | Total N content | 63 | mg/100ml |
|  | Total P content | 300 | mg/l |  | Total P content | 19 | mg/l |

**Table S3.** Comparison of lycopene production cost using different strategies

|  |  | Traditional fermentation | | Self-cycling strategy-1 | | Self-cycling strategy-2 | | Self-cycling strategy-3 | |
| --- | --- | --- | --- | --- | --- | --- | --- | --- | --- |
| Component | Price (USD/kg) | Amount (kg/L) | Cost (USD/L) | Amount (kg/L) | Cost (USD/L) | Amount (kg/L) | Cost (USD/L) | Amount (kg/L) | Cost (USD/L) |
| Fresh water | 0.0005 | 1.000 | 0.00050 | 0.300 | 0.0002 | 0.500 | 0.00025 | 0.500 | 0.00025 |
| *D*-galactose | 35 | 0.020 | 0.70 | 0.006 | 0.21 | 0.010 | 0.35 | 0.012 | 0.42 |
| Uracil | 33 | 0.00005 | 0.00167 | 0.00004 | 0.0014 | 0.00004 | 0.0014 | 0.00004 | 0.0014 |
| Yeast extract | 7 | 0.060 | 0.42 | 0.008 | 0.06 | 0.012 | 0.08 | 0.030 | 0.21 |
| Ethanol | 1.03 | 0.120 | 0.12 | 0.120 | 0.12 | 0.120 | 0.12 | 0.120 | 0.12 |
| Glucose | 0.40 | 0.200 | 0.08 | 0.200 | 0.08 | 0.200 | 0.08 | 0.200 | 0.08 |
| Enzyme | 50.00 | 0.000 | 0.00 | 0.001 | 0.05 | 0.001 | 0.05 | 0.001 | 0.05 |
| Cost/per liter |  |  | 1.33 |  | 0.52 |  | 0.69 |  | 0.89 |
| Material cost cut |  |  |  |  | -61.1% |  | -48.0% |  | -33.2% |
| Other cost |  |  | 2.66 |  | 2.66 |  | 2.66 |  | 2.66 |
| COD/batch (mg/L) |  | 24860 |  | 4972 |  | 14916 |  | 14916 |  |

Traditional fermentation: Fermentation medium composition (w/v): 100% fresh water, 2% *D*-gal, 6% yeast extract, 0.005% uracil, 12% ethanol, 20% glucose

Self-cycling strategy-1: Fermentation medium composition (w/v): 70% wastewater, 30% fresh water, 0.6% *D*-gal, 0.8% yeast extract, 0.004% uracil, 12% ethanol, 20% glucose

Self-cycling strategy-2: Fermentation medium composition (w/v): 50% wastewater, 50% fresh water, 1% *D*-gal, 1.2% yeast extract, 0.004% uracil, 12% ethanol, 20% glucose

Self-cycling strategy-3: Fermentation medium composition (w/v): 50% wastewater, 50% fresh water, 1.2% *D*-gal, 3.0% yeast extract, 0.004% uracil, 12% ethanol, 20% glucose
